# Analysis of Rapid Curing Characteristics of Modified Epoxy Emulsified Asphalt Mixture with Steel Slag Addition Under Microwave Radiation

**DOI:** 10.3390/ma19091880

**Published:** 2026-05-02

**Authors:** Guoqing Gu, Kaijian Huang, Yan Ding, Guomin Wu, Pengyang Song

**Affiliations:** 1 College of Civil Engineering, Nanjing Forestry University, 159# Longpan, Nanjing 210037, China; guoqing10120711@163.com (G.G.); yanding858@163.com (Y.D.); songpengyang0823@163.com (P.S.); 2 Jiangsu Highway Intelligent Detection and Low-Carbon Maintenance Engineering Research Center, Nanjing Forestry University, 159# Longpan, Nanjing 210037, China; 3 Institute of Chemical Industry of Forest Products, Chinese Academy of Forestry, 16# Suojinwucun, Nanjing 210042, China; woogm@hotmail.com

**Keywords:** rapid curing, modified epoxy emulsified asphalt mixture, steel slag, microwave radiation

## Abstract

To address the slow curing and low early strength of conventional modified epoxy emulsified asphalt repair materials, this study introduced steel slag aggregate into epoxy emulsified asphalt mixtures. Experimental techniques including heat absorption–heat transfer rate tests, Marshall stability tests, COMSOL numerical simulation, and scanning electron microscopy (SEM) were adopted to analyze rapid and uniform heating under microwave radiation. The influence of steel slag’s chemical composition, content, and particle size on epoxy curing, asphalt demulsification, and early strength of the mixture was systematically examined. Results show that steel slag containing Fe and Mg elements exhibits higher microwave absorption efficiency. When its content exceeds 15%, the heating rate increases by approximately 0.335 °C/s under the tested conditions. Particles sized 0.6~2.36 mm show better wavelength matching with the applied microwave frequency (2.45 GHz), thereby enhancing absorption. After 140 s of microwave radiation, the core temperature of the mixture reaches 110 °C, which is the appropriate temperature to achieve rapid epoxy curing and synchronous asphalt demulsification. These two processes synergistically form a continuous network structure, thereby improving the compactness and initial laboratory Marshall stability of the mixture. Nevertheless, this study has several limitations. The microwave absorption efficiency depends strongly on the specific mineralogy and Fe/Mg content of steel slag, both of which may vary with source. The conclusions are based on laboratory-scale tests under fixed microwave power and mixture proportions. Despite these limitations, the results demonstrate that steel slag can serve as an effective microwave-absorbing component in epoxy emulsified asphalt mixtures, enabling rapid curing and demulsification to accelerate early strength development.

## 1. Introduction

In recent years, significant advancements have been made in road construction in China [1]. However, issues such as frequent overloading of vehicles have contributed to the rapid deterioration of the service performance of many asphalt concrete pavements [2]. This has prompted further development in road maintenance technologies. Currently, the primary methods for repairing asphalt pavement layers are categorized into two main technical approaches: hot-mix repair and cold-mix repair. Hot-mix repair is predominantly employed to address pavement defects such as depressions and rutting [3]. However, this method is mainly limited to the warmer months of summer and autumn, as it often fails to meet material compaction requirements under low temperatures or in humid conditions [4,5]. Furthermore, while hot-mix asphalt is well-suited for large-scale pavement maintenance projects in geographically concentrated areas, its applicability is restricted in small-scale repair operations [6,7]. In contrast, the cold-mix asphalt technique overcomes the challenges posed by low-temperature environments, making it particularly effective for addressing sudden pavement defects. In cases of localized spalling or potholes, this technique allows for rapid emergency repairs, thereby preventing further damage to the affected area [8,9,10].

Currently, cold-mix asphalt materials available on the market can be broadly classified into three categories: solvent-based cold mixes, reactive cold mixes, and emulsion-based cold mixes [11]. Among these, waterborne epoxy emulsified asphalt represents a type of emulsion-based cold mix, formed by blending waterborne epoxy resin (WER) with liquid emulsified asphalt in a specific ratio. Numerous studies have demonstrated that waterborne epoxy resin exhibits excellent compatibility with emulsified asphalt, significantly enhancing the overall performance of pavement mixtures [12,13,14,15,16,17]. However, most of these studies focus on the final performance after full curing, while paying limited attention to the early-stage behavior. While traditional waterborne epoxy emulsified asphalt cold mixes improve mechanical properties through the crosslinked network structure formed by epoxy resin, the curing process is primarily dependent on natural evaporation and heat conduction. This reliance on these processes leads to several drawbacks, including low early strength and an extended traffic opening period, which remain key challenges for the widespread adoption of such materials [18]. These limitations hinder their widespread adoption, especially for rapid pavement repair where quick strength gain is essential.

To overcome the slow curing issue, microwave radiation has been proposed as an alternative energy input. Microwave radiation works through dielectric loss, converting electromagnetic energy into heat and enabling rapid temperature increase [19]. Research by Geymonat et al. demonstrated that microwave radiation technology could extend the fatigue life of pavements by approximately 20%, while simultaneously reducing raw material and energy consumption by about 50% [20]. Additionally, Sun et al. analyzed the surface heating rate of steel-fiber-modified asphalt under microwave radiation, finding that it could reach up to 26 °C/min, showcasing its remarkable healing performance [21]. These studies confirm the potential of microwave technology for asphalt pavement maintenance. However, they primarily focus on conventional or fiber-modified asphalt mixtures, and the application of microwave radiation to epoxy emulsified asphalt systems remains underexplored.

Emulsified asphalt mixtures typically exhibit poor microwave absorption properties, which limits their ability to achieve the rapid curing and formation required for efficient performance. To address this limitation, researchers have explored the incorporation of microwave-absorbing materials to enhance the utilization of microwave electromagnetic energy within the mixtures. Steel slag, which is rich in metal oxides, has been identified as a promising additive due to its ability to rapidly heat under microwave conditions [22,23,24]. For instance, Gallego et al. examined steel slag with a particle size range of 0~2 mm and found a positive correlation between its content and the heating rate of asphalt mixtures [25]. Similarly, Gulisano et al. demonstrated that replacing 9% of limestone aggregate in an asphalt mixture (AC-20) with steel slag increased the heating rate from 1160 °C/(kWh·kg) to 2089 °C/(kWh·kg) [26]. Moreover, the irregular and rough texture of steel slag forms an interlocking skeletal structure with asphalt, thereby increasing the effective contact area, enhancing the asphalt film thickness, and improving structural asphalt proportion. These factors contribute to better interfacial adhesion between the components [27]. Zhang et al. reported that substituting limestone with steel slag improved the long-term creep resistance of the interfacial asphalt without significantly affecting its tensile properties [28]. Collectively, these studies highlight the considerable potential of incorporating steel slag into asphalt mixtures, particularly in enhancing their performance under microwave radiation. Nevertheless, they are largely limited to conventional asphalt binders and have not systematically addressed the combined use of steel slag with reactive systems such as epoxy emulsified asphalt.

The paper addresses the inherent limitations of epoxy emulsified asphalt cold mixes, such as low early strength and extended curing time, by integrating the microwave thermal effect of steel slag with a novel BC-1 cationic epoxy-modified emulsified asphalt. An investigation was conducted into the synergistic interactions of these components under microwave radiation. In contrast to previous studies that primarily focus on enhancing the performance of a single material, this research adopts a comprehensive experimental and numerical approach, including microwave absorption and heat transfer rate measurements, Marshall stability tests, COMSOL-based numerical simulations, and scanning electron microscopy (SEM). Specifically, this work presents three novel contributions. First, it quantitatively elucidates the microwave radiation characteristics of steel-slag-modified epoxy emulsified asphalt mixtures, and systematically reveals the effects of steel slag with 5~30% content, 0.6~2.36 mm particle size, and Fe- and Mg-rich chemical phases on the heating efficiency of the mixtures. Second, it elucidates the synergistic mechanism among microwave-induced heating, epoxy resin curing, and asphalt demulsification. The results show that the core temperature reaches 110 °C after 140 s of microwave radiation, which meets the temperature requirements for epoxy resin curing and asphalt demulsification, thereby forming a continuous network structure. Third, it evaluates the early-strength development of the mixtures under microwave curing conditions, and demonstrates that the proposed mixture effectively overcomes the inherent drawbacks of conventional epoxy emulsified asphalt cold mixes, namely low early strength and prolonged setting time.

Using the aforementioned methods, this study systematically clarifies the influences of steel slag content, particle size, and chemical composition on the epoxy curing behavior, emulsified asphalt demulsification process, and initial Marshall stability of the mixtures. Overall, the combined application of steel slag and modified epoxy emulsified asphalt establishes a novel research framework, and the observed synergistic effects provide a promising technical strategy for addressing the inherent limitations of epoxy emulsified asphalt cold mix materials.

## 2. Materials and Methods

### 2.1. Waterborne Epoxy Resin Emulsion

In this experiment, bisphenol-A epoxy resin (E51) and polyethylene glycol (PEG-4000 Juxing Chemical Co., Ltd. Shandong, China) were combined in a stoichiometric ratio of 2.1:1 and placed into a three-necked round-bottom flask equipped with a magnetic stirrer and a temperature monitoring system. The flask was immersed in a constant-temperature oil bath, and the system temperature was initially adjusted to 90 °C using a programmed heating profile. Stirring continued until the PEG component completely melted. At this stage, potassium persulfate, used as an initiator at 10 wt% of PEG-4000, was added to the mixture. Subsequently, the reaction temperature was raised to 150 °C, and the polycondensation reaction was maintained under these conditions for 3 h.

Upon completion of the reaction, a golden-yellow, transparent liquid product was obtained, exhibiting high viscosity at room temperature (25 ± 2 °C). An equal mass of deionized water was then added to the synthesized emulsifier, and the mixture was subjected to high-speed dispersion at 1000 r/min in a constant-temperature water bath maintained at 50 °C, followed by 30 min of homogenization to prepare the waterborne epoxy emulsion sample.

The properties of the resulting waterborne epoxy resin emulsion were characterized, and the corresponding results are presented in Table 1.

The property specifications of the raw materials employed in the preparation of the waterborne epoxy resin emulsion are provided in Table 2.

### 2.2. Water-Based Epoxy Resin-Modified Emulsified Asphalt

**Figure 1:**
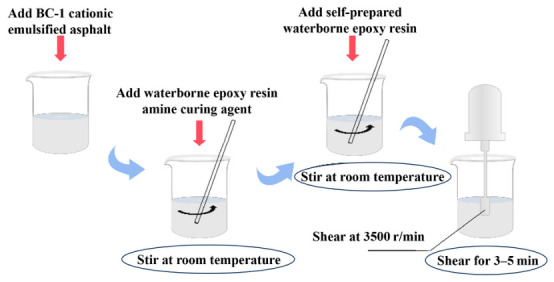
Preparation steps for water-based epoxy emulsified asphalt.

In this experiment, BC-1 cationic emulsified asphalt and the curing agent were initially pre-mixed in Container 1. Container 2 was loaded with a pre-treated epoxy resin emulsion, which accounted for 11% of the mass of BC-1 cationic emulsified asphalt. The two components were then combined and subjected to shear at 3500 r/min for 3~5 min at room temperature (25 ± 2 °C) to obtain the desired waterborne epoxy-modified emulsified asphalt. The detailed procedure is illustrated in Figure 1.

The curing agent is a laboratory-prepared amine curing agent, and the mass ratio of water-based epoxy resin to curing agent is 100:40. The relevant specifications of the emulsified modified asphalt and curing agent used in the experiment are presented in Table 3 and Table 4, respectively.

### 2.3. Aggregates

In this experiment, basalt was employed as the coarse aggregate, while limestone was used as the fine aggregate and mineral filler. The technical specifications of these materials are provided in Table 5.

### 2.4. Steel Slag

In this experiment, X-ray fluorescence (XRF) spectroscopy was used to analyze the chemical compositions of two steel slag samples, designated as SS-1 and SS-2. The results of the analysis are presented in Table 6.

In this experiment, steel slag with particle size distributions of 0.075~0.6 mm, 0.6~2.36 mm, and 2.36~9.5 mm was selected. Through comparative testing, the optimal asphalt content range for the modified epoxy asphalt mixture was identified. The corresponding basic performance parameters are provided in Table 7.

### 2.5. Determination of Gradation

**Figure 2:**
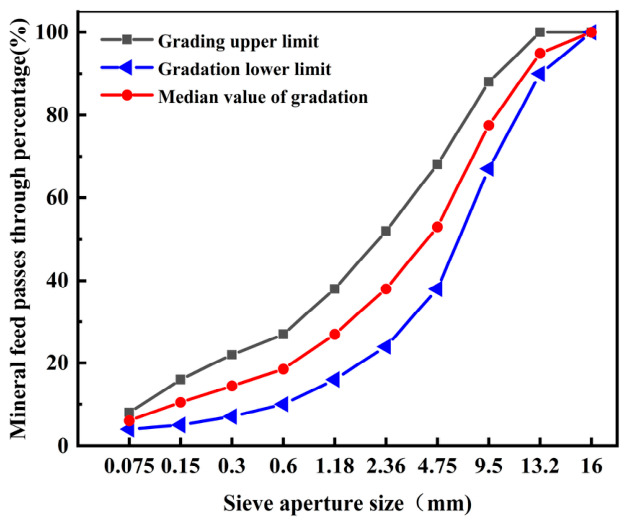
AC-13 gradation curve.

In accordance with the Technical Specifications for Construction of Highway Asphalt Pavements (JTG F40-2004) [31], the gradation design of cold-mixed asphalt mixtures was based on the skeleton-dense balance principle typically applied to hot-mixed asphalt mixtures. In this study, the median gradation of AC-13 was selected as the reference. The corresponding synthetic gradation is presented in Table 8, and the gradation curve is shown in Figure 2.

### 2.6. Mixing Sequence of the Mixture

In this study, the initial emulsion dosage for AC-13 gradation was determined with reference to empirical Formula (1) proposed by the Cationic Emulsified Asphalt Cooperation Group of the Ministry of Transport of China.

*P* = 0.06*A* + 0.12*B* + 0.2*C* (1)

In the Formula (1), *P* is the percentage of emulsified asphalt in the dry mass of mineral aggregates (%), *A* is the mass percentage of mineral aggregates larger than 2.36 mm (%), *B* is the mass percentage of mineral aggregates with particle size of 0.075~2.36 mm (%), and *C* is the mass percentage of mineral filler (%).

Based on the aggregate gradation used in Figure 2, the preliminary emulsion dosage was calculated and determined to be 8.93% according to Equation (1). Then five emulsion dosages of 8.0%, 8.5%, 9.0%, 9.5% and 10.0% were respectively added into the asphalt mixtures. Taking the results of Marshall tests as the response variable, the optimal emulsion dosage was calculated and determined to be 8.59%.

The mixing sequences of the steel slag-modified waterborne epoxy emulsified asphalt mixtures were carried out as follows: steel slag with different particle size ranges was selected according to the gradation curve in Figure 2 to replace part of the aggregate in the original mix proportion design by equal mass, and all materials were placed into an asphalt mixture mixer and mixed at room temperature for 60 ± 5 s. Subsequently, water corresponding to a total water content of 5%, which was determined by former study, was added and mixing was continued at room temperature for an additional 60 ± 5 s. Then, waterborne epoxy emulsified asphalt prepared according to the method described in Section 2.2 of this paper was added at a total emulsion dosage of 8.59% by mass of aggregates, where the waterborne epoxy resin content was 11% by mass of the emulsified asphalt, and mixing was continued at room temperature for 45 ± 5 s. Finally, mineral filler was added and mixing was continued at room temperature for 60 ± 5 s to obtain the target mixture.

### 2.7. Heat Absorption–Heat Transfer Rate Test

**Figure 3:**
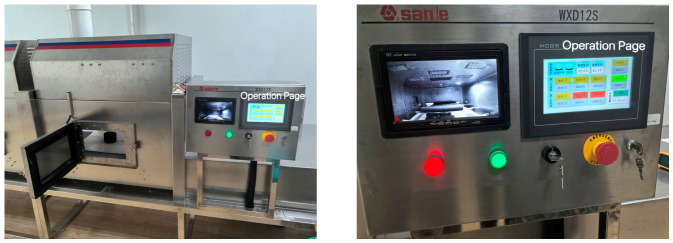
Microwave radiation apparatus for waterborne epoxy emulsified asphalt mixtures.

Two types of steel slag, both within the particle size range of 0.6~2.36 mm, were selected to partially replace aggregates in the original mix design at replacement rates of 5%, 10%, 15%, 20%, 25%, and 30%. The mixtures were initially compacted 35 times on both sides, cured at 25 °C for 24 h, and then subjected to a second double-sided compaction of 45 blows. The resulting specimens were designated as SS-1 and SS-2, respectively. After demolding, the specimens were placed in a microwave radiation apparatus and compared with control specimens without steel slag (SS-0). Microwave radiation was performed under controlled ambient conditions (20 ± 1 °C, relative humidity 50 ± 5 °C). Microwave power was set at 1500 W with a frequency of 2.45 GHz. Prior to the experiments, the apparatus was warmed up for 15 min to ensure stable power output. Surface temperatures were recorded every 20 s with a precision of 0.1 °C. Three parallel specimens were prepared for each test, and the average value of test results was taken. Results are reported as mean ± standard deviation. The experimental heating procedure is illustrated in Figure 3.

### 2.8. Microwave Radiation Simulation Analysis

**Figure 4:**
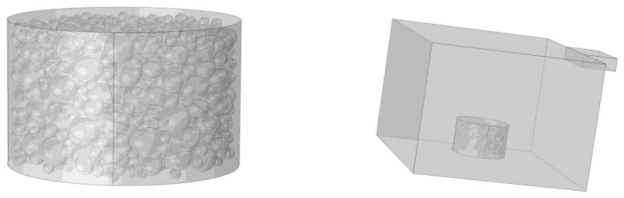
Model of a sample of microwave-heated asphalt mixture.

The coupled microwave thermal effect analysis model established in this study is shown in Figure 4. The model consists of a microwave resonance system and a heating chamber (containing a standard Marshall specimen and a square quartz glass substrate), with structural parameters strictly following the actual experimental setup. The standard Marshall specimen has a diameter of 101.6 mm and a height of 63.5 mm; the quartz glass substrate dimensions are 120 mm × 120 mm × 5 mm.

In the numerical simulation, the energy exchange between the specimen and the environment is considered. The surface heat dissipation process is modeled as a natural convection boundary condition, with the convective heat transfer coefficient h quantifying the surface heat loss. Based on the empirical correlation for natural convection from a vertical flat plate, h is taken as 15 W/(m^2^·K). The following assumptions and limitations are made: (i) local variations in steel slag distribution are ignored because the particles are much smaller than the microwave wavelength, and the effective medium approximation applies; (ii) latent heat of curing and demulsification is neglected, and only sensible heat transfer is considered; (iii) thermal and electromagnetic properties are assumed constant over 20~150 °C, as preliminary measurements indicated variations of <10% for ε′ and <20% for ε″ up to 120 °C.

The material parameters were measured as follows. Thermal properties such as thermal conductivity and specific heat capacity of the mixture were measured at 25 °C using a thermal property analyzer, with an uncertainty of ±5% based on three replicate measurements. Electromagnetic properties such as real/imaginary parts of complex permittivity and complex permeability were measured at 2.45 GHz using a vector network analyzer combined with the waveguide transmission line method. The uncertainties are estimated as ±3% for ε′ and μ′, and ±10% for ε″ and μ″. Frequency dependence was checked by preliminary sweeps from 2.0 to 3.0 GHz and found to be negligible around 2.45 GHz. The measured values of SS-1 are listed in Table 9.

Based on the COMSOL Multiphysics coupling platform, a two-way coupled electromagnetic–thermal numerical model is developed through the coordinated calculation of the electromagnetic wave module and the solid heat transfer module. This model can accurately solve the electromagnetic field intensity distribution and the evolution of the non-uniform temperature field induced during microwave radiation. The implementation procedure is as follows: a 2.45 GHz microwave excitation source is set in the electromagnetic calculation module, a convective heat flux boundary condition is defined in the heat transfer analysis module, and the dynamic conversion from electromagnetic energy to thermal energy is simulated using a two-way field-coupling algorithm. The coupling algorithm sequentially solves Maxwell’s equations for the electric field distribution E, computes the volumetric heat source by Formula (2).

(2) $$Q=\frac{1}{2}\omega \varepsilon_{0}\varepsilon^{\prime \prime }{|\;E\;|}^{2}$$

In the Formula (2), ω is the angular frequency, $\varepsilon_{0}$ the vacuum permittivity, and $\varepsilon^{\prime \prime }$ the imaginary part of the relative permittivity. Then solves the heat transfer equation; the updated temperature field feeds back to modify the material properties, achieving full coupling. Adaptive meshing in COMSOL is used for the calculation, with a maximum element size of λ/10≈12 mm in the specimen region, refined to λ/20 near edges. A mesh convergence test shows that further refinement changes the maximum temperature by less than 0.5 °C.

To evaluate model robustness, a sensitivity analysis was performed by varying key input parameters (thermal conductivity k, specific heat $c_{p}$, dielectric loss $\varepsilon^{\prime \prime }$, and convective coefficient h) by ±20%. The resulting changes in core temperature after 140 s are: $\Delta T_{k}=\pm 2.1\;{}^{\circ}C$, $\Delta T_{c_{p}}=\pm 3.2\;{}^{\circ}C$, $\Delta T_{\varepsilon^{\prime \prime }}=\pm 4.5\;{}^{\circ}C$, $\Delta T_{h}=\pm 1.8\;{}^{\circ}C$. These variations are within the experimental temperature measurement uncertainty (±1.2 °C), confirming that the model predictions are not overly sensitive to parameter uncertainties.

**Figure 5:**
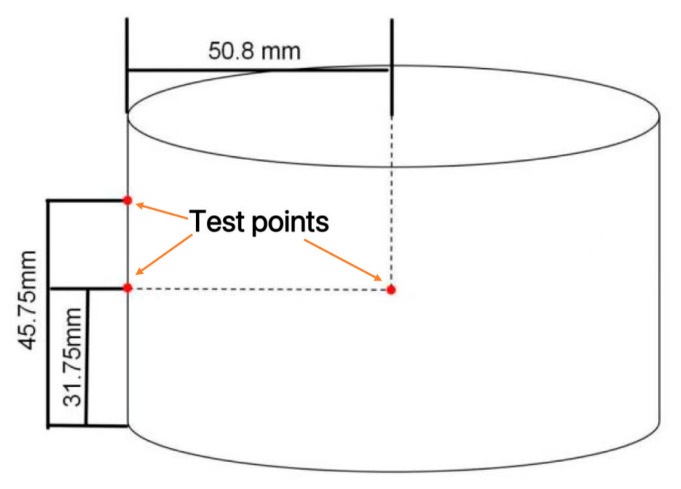
Schematic diagram of internal and external temperature test points.

Finally, the model was validated by extracting temperatures at the coordinates shown in Figure 5 from the simulation at heating times of 40, 60, 80, 100, 120, and 140 s and comparing them with experimental measurements. The mean absolute deviation is 2.5 °C, which is within the combined uncertainty.

### 2.9. Marshall Stability Test

**Figure 6:**
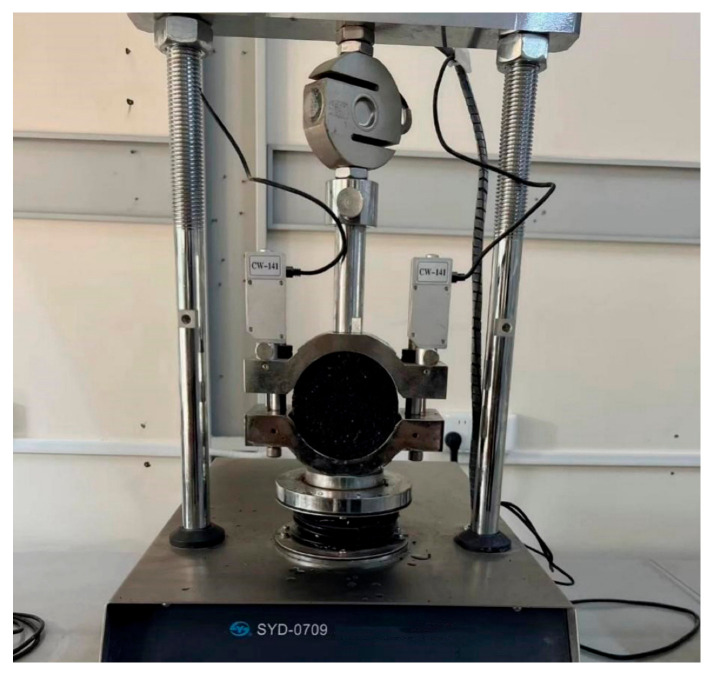
Marshall stability tester.

The molded specimens were first conditioned in a 60 °C water bath for 30~40 min, and then tested using a Marshall apparatus. Vertical loading was applied at a rate of 50.8 mm/min until specimen failure, with the maximum load recorded. Three parallel specimens were prepared for each test, and the average value of the test results was taken. The testing procedure is illustrated in Figure 6.

## 3. Results

### 3.1. Effect of Steel Slag on the Heating Rate of Water-Based Epoxy Emulsified Asphalt Mixture

#### 3.1.1. Effect of Steel Slag Type and Dosage on Absorption–Heat Transfer Rate

During microwave radiation of waterborne epoxy emulsified asphalt mixtures containing steel slag, the dielectric and magnetic loss properties of the slag vary according to its internal chemical composition. As a result, the type of steel slag exerts a significant influence on the microwave absorption capacity and heating rate of the mixture. Furthermore, the dosage of steel slag affects the overall microwave absorption efficiency. When the slag content exceeds a critical threshold, uneven particle distribution within the mixture may induce an electromagnetic shielding effect, causing excessive surface heating while limiting the temperature rise in the interior. In this study, SS-1 and SS-2 steel slags with a particle size of 0.6~2.36 mm were selected for comparative testing.

**Figure 7:**
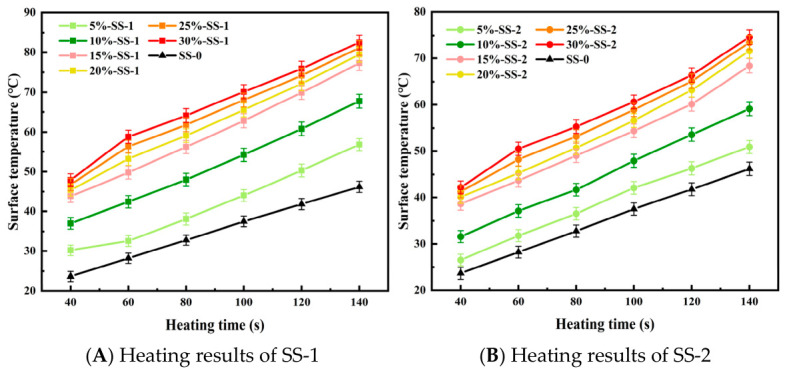
Microwave radiation results of (**A**) SS-1 and (**B**) SS-2 with the same particle size at different dosages.

Figure 7 illustrates that steel slag of different types but identical particle sizes exhibits distinct surface temperatures when incorporated into asphalt mixtures at equivalent proportions. Compared with the control group (SS-0) without slag addition, the surface temperature of the emulsified asphalt mixture increased significantly upon incorporation of steel slag. A comparison between mixtures containing SS-1 and SS-2 steel slag indicates that, at the same addition rate, the mixture with SS-1 achieves a substantially higher surface temperature than that with SS-2. This difference can be attributed to the higher concentrations of Fe and Mg in SS-1, which enhance its microwave absorption efficiency. Consequently, microwave energy is more effectively converted into thermal energy, rendering SS-1 a more suitable microwave-absorbing material for waterborne epoxy emulsified asphalt mixtures [32].

Moreover, the results indicate that the surface temperature of the asphalt mixture increases continuously with the addition of steel slag, reaching a maximum of 82.5 °C. The heating rate of the mixture surface also rises with increasing steel slag content, attaining a maximum of 0.346 °C/s at 30% slag content; however, the rate of increase gradually diminishes. This behavior can be explained by the relatively uniform distribution of steel slag particles at lower contents, which facilitates efficient heat conduction throughout the mixture and results in a rapid rise in surface temperature. When the slag content exceeds 15%, particle agglomeration reduces uniformity, obstructs heat transfer pathways, and slows the overall temperature increase in the mixture. Beyond a certain content threshold, the number of steel slag particles capable of effectively absorbing microwave energy approaches saturation, such that further increases in slag content yield only marginal improvements in absorption efficiency. Additionally, when the slag particles form an excessively dense structure within the mixture, microwave penetration depth is reduced, limiting the effect of electromagnetic waves to the surface or near-surface layers. This leads to the accumulation of heat near the surface, thereby influencing the overall heating rate of the mixture.

#### 3.1.2. Effect of Steel Slag Particle Size on Absorption–Heat Transfer Rate

**Figure 8:**
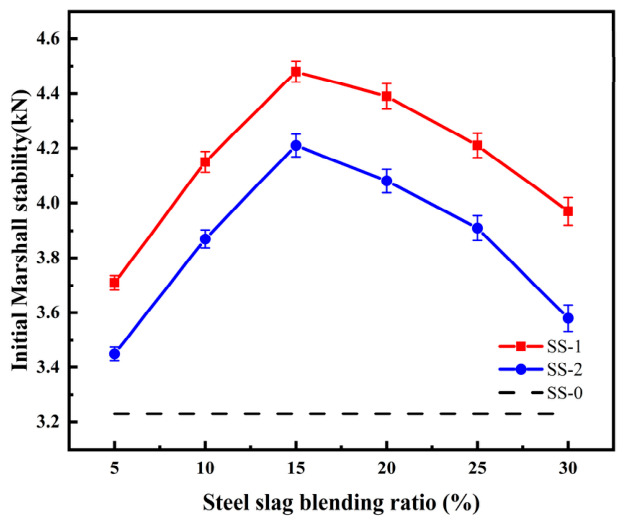
Microwave radiation curves of SS-1 with different particle sizes at 15% content.

The influence of steel slag particle size on the microwave radiation behavior of waterborne epoxy emulsified asphalt mixtures was also investigated. Heating tests were performed using SS-1 steel slag of varying particle sizes, and the resulting temperature–time curves are presented in Figure 8.

The results indicate that, following variation in the incorporated steel slag particle size, both the surface temperature and heating rate under microwave radiation are higher than those observed for asphalt mixture specimens without steel slag. Nevertheless, the efficiency of microwave radiation differs notably among steel slags of varying particle sizes.

Specifically, the specimen incorporating steel slag with a particle size of 0.6~2.36 mm achieved a surface temperature of 77.3 °C after 140 s of microwave radiation, corresponding to a heating rate of 0.335 °C/s, and exhibited the most efficient heat absorption. Specimens containing steel slag in the 2.36~9.5 mm range ranked second in performance, while those with 0.075~0.6 mm particles showed the poorest heating behavior. This performance can be attributed to the dependence of microwave radiation efficiency on the internal electromagnetic field distribution and its compatibility with the microwave wavelength. When the particle size of steel slag falls within the 0.6~2.36 mm range, the dimensions of the particles match favorably with the microwave wavelength, promoting efficient electromagnetic field distribution and internal reflection, thereby enhancing energy absorption and heating rate. Although larger steel slag particles (2.36~9.5 mm) contain higher amounts of magnetic oxides such as iron oxides, which can enhance microwave absorption, the wider interparticle gaps may induce a thermal shielding effect, leading to uneven heat distribution and impeding internal heat transfer. In contrast, moderately sized particles (0.6~2.36 mm) are more uniformly distributed within the mixture, facilitating rapid and even heat transfer and minimizing localized overheating or heat stagnation. Conversely, excessively large or fine particles disrupt uniform heating, ultimately reducing the overall heating rate of the mixture.

### 3.2. COMSOL Simulation of Microwave Radiation Analysis

**Figure 9:**
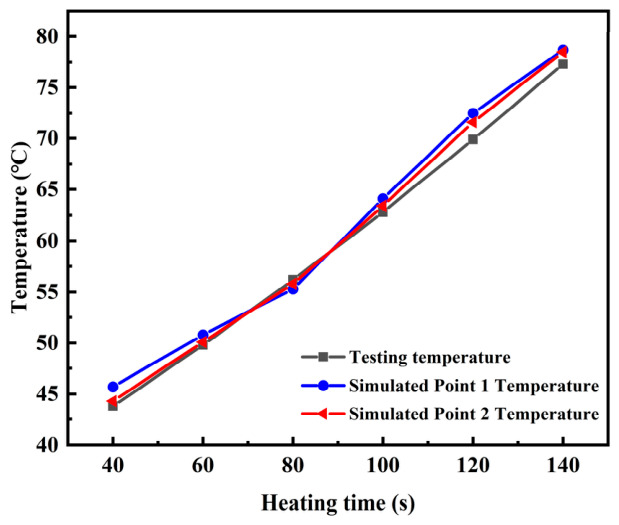
Simulated test points temperature and measured temperature curves.

The microwave radiation behavior of the steel slag-modified waterborne epoxy emulsified asphalt mixture was simulated using COMSOL Multiphysics 6.2 software. Figure 9 presents the relationship between surface temperature and heating time, comparing the simulation results with experimentally measured data.

Correlation analysis between the simulated and experimental data shows that the temperature trends presented in Figure 9 are highly consistent, with an overall correlation coefficient of 0.97, a mean absolute error of 2.3 °C, and a root mean square error of 3.1 °C, despite some localized deviations. These discrepancies primarily arise from minor differences between the iron oxides content specified in the model and the actual measured values in the steel slag, which slightly overestimates the dielectric loss in the simulation. Furthermore, in the peripheral regions, the simulated temperatures exceeded the experimental values by approximately 1.4 °C, attributable to the model’s inability to fully represent the actual spatial distribution of steel slag particles, including local agglomeration effects.

Despite these deviations, the model demonstrates acceptable predictive accuracy for key parameters (15% dosage, 0.6~2.36 mm particle size) in engineering applications, exhibiting a mean absolute error of 2.3 °C and a root mean square error of 3.1 °C. These results indicate that the COMSOL model effectively captures the coupled relationship among steel slag content, particle size distribution, and the microwave-induced thermal response.

**Figure 10:**
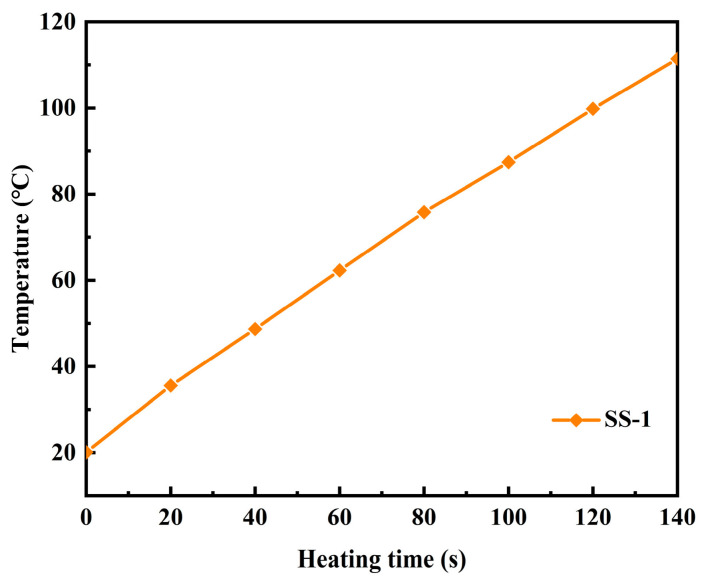
Temperature curve of internal test point. (Simulation result).

As shown in Figure 10, when the waterborne epoxy emulsified asphalt mixture containing 15% SS-1 steel slag with a particle size of 0.6~2.36 mm is subjected to microwave heating for 140 s, the core temperature exceeds 110 °C. This temperature not only satisfies the threshold required for effective curing of the waterborne epoxy resin (>90 °C) but also facilitates an accelerated demulsification rate of the emulsified asphalt.

**Figure 11:**
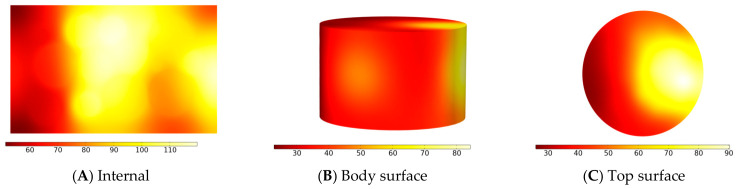
Simulates the temperature gradient of the model’s (**A**) internal section, (**B**) body surface and (**C**) upper top surface of microwave heating at 140 s. (Simulation result).

As shown in Figure 11, under microwave heating, the mixture exhibits a characteristic “high-temperature core and low-temperature periphery” gradient. It is suggested that this phenomenon is associated with the selective microwave absorption behavior of the steel slag particles: magnetic components within the slag efficiently absorb 2.45 GHz microwave energy, transforming the particles into localized heat sources. The generated heat is then rapidly conducted through the steel slag–asphalt interface to regions rich in epoxy resin, potentially accelerating the cross-linking reaction. As microwaves penetrate the mixture, the internal steel slag particles preferentially absorb energy and heat rapidly, whereas the surface region loses heat more readily due to contact with the surrounding air. Moreover, a portion of the microwave energy is continuously consumed by the steel slag during penetration, likely resulting in energy accumulation within the core. The relatively low thermal conductivity of the mixture further impedes the transfer of internally generated heat to the surface, thereby amplifying the temperature difference between the interior and exterior regions.

### 3.3. Effect of Steel Slag on the Initial Marshall Stability of Epoxy Emulsified Asphalt Mixtures

#### 3.3.1. Effect of Steel Slag Type and Dosage on Initial Marshall Stability

**Figure 12:**
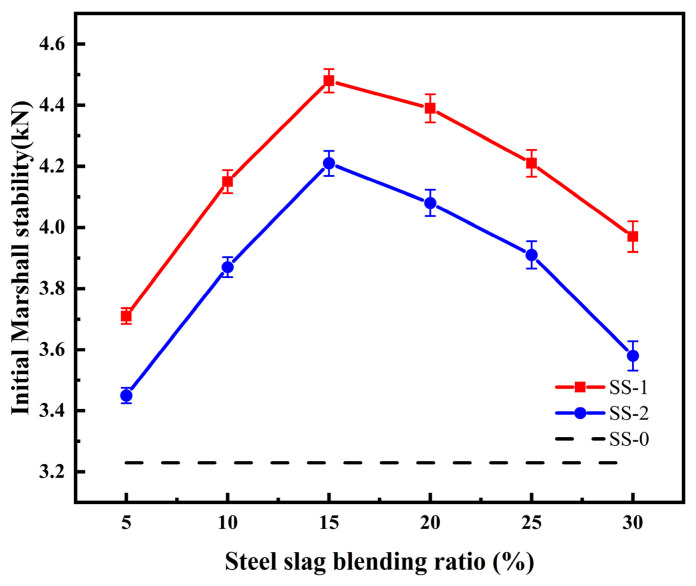
Initial Marshall stability of an asphalt mixture mixed with different steel slags.

Two types of steel slag, SS-1 and SS-2, with particle sizes ranging from 0.6 to 2.36 mm, were selected and incorporated into the waterborne epoxy emulsified asphalt at varying proportions. The initial Marshall stability of the resulting mixtures was subsequently evaluated, and the results are presented in Figure 12.

The results indicate that incorporation of steel slag led to an increase in the initial Marshall stability of the asphalt mixture compared with the SS-0 group (3.23 kN). This improvement is attributed to the partial role of steel slag as an aggregate when replacing the original aggregate in equal proportions, thereby enhancing the mechanical strength of the mixture. In addition, under microwave radiation, steel slag with favorable microwave absorption properties markedly elevates the temperature of the asphalt mixture. Consequently, the waterborne epoxy resin within the mixture undergoes more rapid and effective curing, forming a network-like interconnected structure that densifies the internal framework of the asphalt mixture, enhances the viscoelasticity of the asphalt matrix, and further improves its mechanical performance [33].

Following the incorporation of steel slag, the initial Marshall stability of the asphalt mixture initially increased with increasing slag content, reaching a maximum improvement of 38.7%, and subsequently decreased at higher dosages, with the rate of enhancement gradually diminishing. This behavior is primarily attributed to the gradual reduction in the mechanical contribution of steel slag as an aggregate with increasing content. It is suggested that when the steel slag content exceeds 15%, the original gradation of the asphalt mixture may be disrupted, potentially leading to gradation imbalance and a slight reduction in initial Marshall stability. Additionally, excessive steel slag content may lead to particle agglomeration, which limits the penetration of electromagnetic waves into the mixture, thereby reducing overall heating efficiency and weakening the synergistic interaction between the epoxy system and the emulsified asphalt. Consequently, further increases in slag content provide limited additional strength enhancement. These findings are consistent with the typical response of mixtures in a microwave radiation regime.

#### 3.3.2. Effect of Steel Slag Particle Size on Initial Marshall Stability

**Figure 13:**
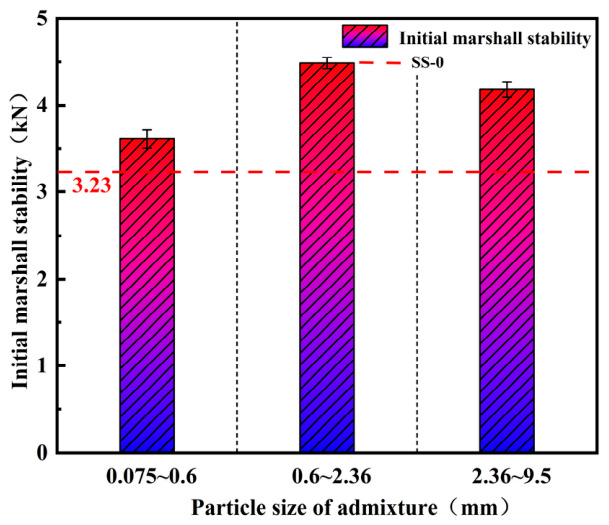
Initial Marshall stability of SS-1 with different particle sizes at 15% content.

Initial Marshall stability tests were conducted on specimens incorporating SS-1 aggregate of varying particle sizes, and the corresponding results are presented in Figure 13.

The results indicate that, regardless of the steel slag particle size, the initial Marshall stability of all specimen groups was higher than that of the asphalt mixture without steel slag. Among them, steel slag with a particle size range of 0.6~2.36 mm produced the most significant enhancement, increasing the mixture strength by approximately 38.7%. This can be attributed to the fact that, once the initial gradation of the asphalt mixture was established, steel slag with different particle sizes exerted varying degrees of skeletal reinforcement when used as aggregate replacements. When finer steel slag particles were used to replace 15% of the aggregate, the original gradation of the mixture was substantially altered, leading to a weakened internal skeleton. Consequently, specimens incorporating finer steel slag exhibited only an approximately 11.8% increase in initial Marshall stability, indicating a relatively limited strengthening effect. In contrast, steel slag with larger particle sizes introduces wider interparticle voids, which may induce a thermal shielding effect that reduces microwave radiation efficiency. This, in turn, adversely affects the curing of the epoxy system and the demulsification of the emulsified asphalt. As a result, the improvement in initial Marshall stability for specimens containing larger steel slag particles is less pronounced than that achieved with steel slag in the 0.6~2.36 mm range.

### 3.4. Comparison of SEM Images Before and After Microwave Radiation

Scanning electron microscopy (SEM) was employed to characterize the microstructural features of the mixture before and after microwave radiation, with particular emphasis on the evolution of the skeletal structure of the waterborne epoxy resin and the emulsified asphalt. Small blocks of approximately 5 mm × 5 mm × 5 mm were cut from the core region of the epoxy emulsified asphalt mixtures before and after microwave radiation. The samples were placed in a vacuum drying oven at 40 ± 2 °C for 24 h to remove residual moisture. After drying, the samples were attached to aluminum stubs using conductive carbon tape and sputter-coated with gold using an ion sputter coater. Observations were performed using a field-emission scanning electron microscope (SEM, model: Zeiss Sigma 360, Shenzhen, China) at an accelerating voltage of 5 kV, working distance of 8~12 mm, and magnifications of 500× and 5000×.

**Figure 14:**
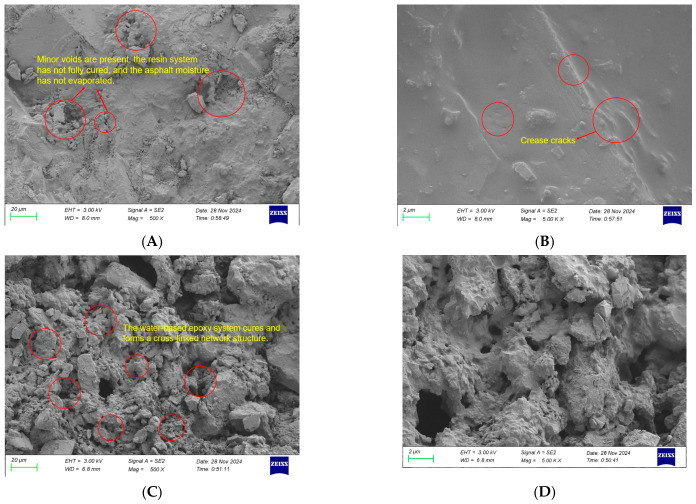
SEM image of waterborne epoxy resin emulsified asphalt mixture before (**A**,**B**) and after (**C**,**D**) microwave radiation. (**A**) SEM images of the sample before microwave radiation, magnification: 500×. (**B**) SEM images of the sample before microwave radiation, magnification: 5000×. (**C**) SEM images of the sample after microwave radiation, magnification: 500×. (**D**) SEM images of the sample after microwave radiation, magnification: 5000×.

The corresponding results are presented in Figure 14.

As shown in Figure 14A,B, before the microwave radiation, only a limited number of pores are observed in the mixture. This is attributed to the incomplete self-curing reaction of the epoxy resin, which has not yet developed a spatial skeletal structure. In addition, the asphalt on the specimen surface exhibits localized collapse, resulting in an uneven morphology characterized by folds and cracks, with few observable pores.

In contrast, Figure 14C,D demonstrate that, after microwave radiation, the emulsified asphalt mixture exhibits a higher pore density and a noticeably rougher surface. This behavior arises from the conversion of microwave energy into thermal energy by microwave-absorbing components, leading to a rapid temperature increase. The elevated temperature promotes the formation of a three-dimensional network structure through the curing of the waterborne epoxy resin within the emulsified asphalt. Simultaneously, thermal-induced demulsification of the emulsified asphalt triggers interfacial linking reactions within the system, causing the continuous phase to transition from asphalt to epoxy resin [34,35]. Moreover, owing to its inherent mechanical strength, the cured epoxy resin is capable of forming a continuous skeletal framework within the asphalt matrix [36,37,38]. These observations further confirm that microwave radiation effectively accelerates emulsified asphalt demulsification and enhances the curing process of the waterborne epoxy resin, thereby contributing to the development of a stable internal structure.

## 4. Conclusions

In this experiment, a self-prepared waterborne epoxy resin was used to modify emulsified asphalt. Steel slag materials with specified particle sizes and proportions were incorporated into the mixture. The microwave thermal effect of steel slag was utilized to enable the system to rapidly reach the temperatures required for the curing of the waterborne epoxy resin system and the demulsification of the emulsified asphalt, accelerate the reaction processes, and thereby enhance the initial laboratory Marshall stability of the mixture. Analyses of skeletal structure changes in water-based epoxy resin and emulsified asphalt before and after microwave radiation were performed via scanning electron microscopy (SEM). By integrating the microwave thermal effect of steel slag with the curing characteristics of the waterborne epoxy resin, the following conclusions were drawn:

(1) SS-1 steel slag exhibits a significantly higher microwave absorption rate than SS-2 steel slag due to its higher content of magnetic oxides such as Fe and Mg. Under identical conditions of steel slag content and particle size range, the temperature of specimens containing SS-1 steel slag was approximately 13% higher than that of SS-2 specimens after 140 s of microwave radiation. As the steel slag content increased, the surface temperature of the asphalt mixture rose accordingly. However, when the slag content exceeded 15%, the distance between steel slag particles decreased and local aggregation occurred, resulting in reduced uniformity of heat transfer and obstruction of thermal conduction pathways. Consequently, the overall heating rate of the mixture tended to plateau. Once the steel slag content exceeded a certain threshold, the amount of slag effectively absorbing microwave energy approached saturation, and further increases in content led to only marginal improvements in microwave absorption efficiency. With respect to particle size selection, when the steel slag particle size ranged from 0.6 to 2.36 mm, the particle dimensions more effectively matched the microwave wavelength, yielding the optimal heating rate of 0.335 °C/s. In contrast, excessively large or small particle sizes weakened performance due to non-uniform heat distribution or a loosened structural skeleton. Based on these results, SS-1 steel slag with a particle size range of 0.6~2.36 mm and a content of 15% was selected, at which point the overall temperature of the mixture fully met the requirements for curing of the waterborne epoxy system and demulsification of the emulsified asphalt.
(2) COMSOL simulation results indicate that when the waterborne epoxy emulsified asphalt mixture is uniformly filled with fine steel slag exhibiting superior microwave absorption properties, a more uniform and favorable temperature distribution is achieved. Moreover, the central temperature of the asphalt mixture is consistently higher than the surface temperature, fully satisfying the temperature requirements for curing of the waterborne epoxy system and demulsification of the emulsified asphalt. By comparing the simulation results with the experimentally measured data, it can be concluded that when the content of steel slag with a particle size range of 0.6~2.36 mm is 15%, COMSOL effectively and accurately simulates the temperature distribution of waterborne epoxy emulsified asphalt mixtures incorporating steel slag under microwave radiation.
(3) Marshall stability tests indicate that steel slag converts electromagnetic energy into thermal energy, thereby accelerating the curing of the waterborne epoxy system and the demulsification of the emulsified asphalt. As a result, the initial laboratory Marshall stability after microwave radiation of specimens incorporating steel slag is significantly higher than that of specimens without steel slag. When steel slag with a particle size range of 0.6~2.36 mm is incorporated at a content of 15%, the initial Marshall stability values of the SS-1 and SS-2 specimens reach 4.48 kN and 4.21 kN, respectively, representing increases of 38.7% and 30.3% compared with the control group. However, with further increases in steel slag content, the original gradation of the asphalt mixture is disrupted, and the mechanical contribution of steel slag as an aggregate gradually diminishes, leading to a reduction in strength. When steel slag with the same particle size range is incorporated at equal proportions, the initial Marshall stability of the SS-1 specimens is generally about 7% higher than that of the SS-2 specimens. Steel slag particles in the size range of 0.6~2.36 mm exhibit better matching with the microwave wavelength, resulting in optimal initial Marshall stability. In contrast, excessively large or small particle sizes weaken performance due to non-uniform heat distribution or a loosened structural skeleton.
(4) SEM analysis reveals that, before microwave radiation, only a limited number of pores are present in the mixture, and the self-curing reaction of the epoxy resin is incomplete, failing to form a spatial skeletal structure. After microwave radiation, the waterborne epoxy resin develops a continuous network skeleton, while the asphalt uniformly coats the aggregates with a tightly bonded interface. The elevated temperature accelerates the synergistic processes of emulsified asphalt demulsification and epoxy resin curing. Through the “microwave absorption–heat transfer–rapid curing” pathway, the “asphalt–steel slag–epoxy” three-phase composite structure is effectively reinforced, ultimately enhancing the compactness and mechanical properties of the mixture.
(5) The microwave-assisted technique utilizing steel-slag-modified epoxy emulsified asphalt is well-suited for field pavement repair. Specifically, according to the COMSOL simulation, mobile microwave units can elevate the core temperature of the mixture to 110 °C within 140 s, which is predicted to facilitate rapid curing and demulsification, thereby potentially shortening the traffic opening time. Nevertheless, achieving uniform microwave distribution over large repair areas and optimizing the design of microwave applicators remain prominent engineering challenges that require further exploration.

The incorporation of steel slag—an industrial by-product—offers notable environmental and economic benefits: it reduces the volume of waste sent to landfills, minimizes the extraction of natural aggregates, and lowers material costs, particularly in regions with steel production industries. Additionally, compared with traditional hot-mix asphalt methods, microwave-accelerated curing technology further reduces energy consumption, enhancing the sustainability of the repair process.

It should be noted that this study focused primarily on the early-strength development of the mixture. Long-term performance indicators, including durability, fatigue resistance, and moisture susceptibility, were not investigated in the present work and thus warrant thorough examination in future research.

## Figures and Tables

**Table 1:** Waterborne epoxy resin specifications.

Appearance	Test Value	Test Method
Physical form	Milky-white viscous liquid	Visual inspection
Solid content	50 ± 2	GB/1725-1979 [29]
Epoxy value	0.08 mol/100 g	Hydrochloric acid-acetone method
Viscosity	1000–2000	DV-type rotational viscometer
pH	6~7	pH meter
Specific gravity (g/cm^3^)	1.05 ± 0.05	GB1756-1979 [30]
Mean particle size/μm	1.17	Laser particle size analyzer
Storage stability	>360 d	Self-measurement

**Table 2:** Water-based epoxy resin raw materials.

Raw Materials	Manufacturer	Remarks
Bisphenol A Epoxy Resin E51	Yoshida Chemical Co., Ltd. Shenzhen, China	Viscous liquid, industrial product
Potassium persulfate	Aladdin Biochemical Technology Co., Ltd. Shanghai, China	Molecular formula: K_2_S_2_O_8_ Molecular weight: 270.3 Analytical grade
Polyethylene glycol	Juxing Chemical Co., Ltd. Shandong, China	Molecular weight range: 3600~4400 Melting point: 55 °C pH: 6~8

**Table 3:** Performance indexes of emulsified asphalt.

Demulsification Rate	Particle Charge	Road Standard Viscosity C_25.3_/S	Evaporation Residue		
			Penetration (25 °C)/0.1 mm	Softening Point/°C	Ductility (15 °C)/cm
Slow fracture	Cation (+)	12.3	73	53	118

**Table 4:** Technical parameters of curing agent.

Appearance	Pale Yellow Uniform Liquid
Solid content/%	45 ± 2
Solids/%	22~28
VOC content/%	<2
pH	9~10
Particle size/μm	<2

**Table 5:** Performance indexes of aggregates and mineral fines.

Types	Technical Indicators	Test Value	Specified Requirement Value
Coarse aggregate	Particle Size Range/mm	2.36~9.5	2.36~9.5
	Apparent Relative Density	2.718	≥2.60
	Stone Crushing Value/%	15	≤26
	Los Angeles Abrasion Loss/%	20	≤28
	Stone polish value/BPN	50	≥42
	Sturdiness/%	7	≤12
	Needle and flake content/%	10	≤15
Fine aggregate	Particle Size Range/mm	0.075~2.36	0.075~2.36
	Apparent Relative Density	2.723	≥2.50
	Silt content/%	0.76	≤3
	Sand equivalent/%	70	≥60
	Angularity/s	34	≥30
Mineral powder	Apparent Relative Density	2.726	≥2.50
	Moisture content/%	0.76	≤1
	Hydrophilicity coefficient	0.8	<1
	Plasticity Index	2.5	<4

**Table 6:** Main chemical components of steel slag.

Type	O	Si	Ca	Fe	Mg
	Mass Fraction/%	Mass Fraction/%	Mass Fraction/%	Mass Fraction/%	Mass Fraction/%
SS-1	24.22	2.41	15.6	35.37	13.60
SS-2	29.51	9.87	31.63	12.52	6.51

**Table 7:** Performance indexes of slag.

Technical Indicators	Test Value	Specified Requirement Value
Apparent Relative Density	3.3	≥2.90
Water-soak expansion rate/%	0.9	≤2.0
Water absorption rate/%	1.2~2.5	≤3.0
Crush value/%	12.8	≤26
Los Angeles Abrasion Loss/%	12.7	≤26

**Table 8:** Grading range of AC-13 asphalt mixture.

Sieve Size (mm)	16.0	13.2	9.5	4.75	2.36	1.18	0.6	0.3	0.15	0.075
Maximum gradation limit	100	100	88	68	52	38	27	22	16	8
Minimum gradation limit	100	90	67	38	24	16	10	7	5	4
Median gradation	100	95	77.5	53	38	27	18.5	14.5	10.5	6

**Table 9:** Thermal parameters and electro-magnetic properties of steel slag emulsified SS-1 asphalt mixture.

Thermal Conductivity (k) [W/(m·K)]	Specific Heat (C) [J/(g·K)]	Real Part of the Dielectric Constant (ε′)	Imaginary Part of the Dielectric Constant (ε″)	Real Part of Magnetic Permeability (μ′)	Imaginary Part of the Magnetic Permeability (μ″)
1.054	1.113	3.769	0.074	1.058	0.027

## Data Availability

The original contributions presented in this study are included in the article. Further inquiries can be directed to the corresponding author.

## Abbreviations

The following abbreviations are used in this manuscript:

WER	Waterborne Epoxy Resin
